# Gender Difference and Correlates of Physical Activity Among Urban Children and Adolescents in Ethiopia: A Cross-Sectional Study

**DOI:** 10.3389/fpubh.2022.731326

**Published:** 2022-03-08

**Authors:** Sibhatu Biadgilign, Bereket Gebremichael, Admas Abera, Tsedey Moges

**Affiliations:** ^1^Public Health Nutrition Research Consultant, Addis Ababa, Ethiopia; ^2^College of Health Sciences, Addis Ababa University, Addis Ababa, Ethiopia; ^3^Department of Epidemiology and Biostatistics, School of Public Health, Haramaya University, Harar, Ethiopia; ^4^Food Science and Nutrition Research Directorate, Ethiopian Public Health Institute, Addis Ababa, Ethiopia

**Keywords:** physical activity and exercise, childhood, overweight/obesity, Ethiopia, gender difference

## Abstract

**Background:**

Studies indicate that children and adolescent populations in most countries show a low level of physical activity (PA) and an increasing prevalence of obesity. Addressing gender disparity in PA is the main element of public health programs. There is currently a paucity of studies, particularly, in developing countries that investigate gender differences and correlates of PA among children and adolescents.

**Objective:**

The study is aimed to assess the gender difference and correlates of PA among children and adolescents in Ethiopia.

**Methods:**

An observational population-based cross-sectional study was conducted in representative samples of children and adolescents in the capital city of Ethiopia, Addis Ababa. Multivariable logistic regression models with robust estimation of SEs were fitted to predict the odds ratios (ORs) and 95% CIs.

**Results:**

A total of 632 children and adolescents-parent dyads were included in the study. More boys than girls (17.0 and 11.7%) were engaged in moderate intensity PA 3 days a week or more (*p* = 0.057). Age, mothers working in a private business, attending public schools, longer sleep duration, and being taught the benefits of PA were positively associated with meeting moderate-to-vigorous PA (MVPA) in both sexes combined and in a sub-sample of boys. Furthermore, an inverse association was found between overweight/obesity and MVPA in the overall children and girls as well. For moderate PA (MPA); the age of the children, maternal education and occupation, school type, overweight/obesity, and sleep duration on school nights were significant correlates among the studied children.

**Conclusions:**

The present study provided evidence of several correlates identified associated with meeting MVPA and MPA in both sexes combined. Girls are less likely than boys to engage in PA. Therefore, there is a need to take into perspectives the provision of a comprehensive multifaceted health behavior modification and interventions, such as focused and regular physical education in schools.

## Introduction

Globally, the prevalence of insufficient physical activity (PA) among school-attending adolescents was 78.4% for boys and 84.4% for girls aged 11–17 years (1). Studies also indicate that children and adolescent populations in most of the developing countries exhibit a low prevalence of overall PA levels and a rising prevalence of obesity (1). PA for children includes active play, walking or biking, exercising, school-based activities, recreational activities, etc. Identifying and addressing gender disparity in PA is the main element of public health programs (2). The health benefits of being physically active during adolescence are well-documented (3). PA is associated with a marked reduction in premature mortality and reduces risks of more than 24 chronic diseases, such as cardiovascular diseases, diabetes, and cancers (4, 5).

Different literature found out that girls were lower perceived competence in physical education (6) and were less active than boys and less physically fit compared to boys (1, 6–9). In a large scale, about 219,803 participants from 33 out of 47 Latin-American countries from 5 to 17 years study, in general, boys showed a higher prevalence of meeting PA guidelines in comparison with girls (10) and from 2009 to 2010 Health Behavior in School-Aged Children study included 36 countries indicate that boys reported more PA than girls, but the magnitude of these sex differences varied greatly between countries (11).

There is evidence to suggest that many children and adolescents do not engage in healthy behaviors at the recommended levels with multiple obesity risk behaviors (12). Findings from a review suggest that obesogenic cluster patterns are complex with a mixed PA/sedentary behaviur cluster observed most frequently. The tendency for older children/adolescents, particularly, girls to comprise clusters defined by low PA was also a robust finding (13).

In recent years, there are increased opportunities for children to become sedentary in their leisure time. Particularly, technology has offered more opportunities to play video games, watch TV, and browse the internet that are shown to be positively related with overweight and time spent on sedentary behaviors is inversely associated with physical exercise among adolescents (14, 15). The reduced level of PA in children is greatly associated with unfavorable metabolic and cardiovascular outcomes (16), reduced well-being, and cognitive function (17). Hence, this entails there is a need to promote PA in children across a spectrum of environmental settings (18, 19).

According to a recent review by Martin et al. (20), there is a growing body of literature that shows the influence of lifestyle interventions with modified forms of PA and dietary management that can impact the body's energy balance and metabolic system. School physical education is recognized as a key opportunity for improving PA by providing children and adolescents fundamental knowledge, movement skills, and active attitude for lifetime PA (12, 13, 21).

In Ethiopia, to address the problem of obesity, the National Nutrition Program incorporated the importance of PA to reduce obesity and its complications (22). However, the efforts do not target children and adolescents well. Although studies on PA have been conducted in Ethiopia, the risk factors for physical inactivity among children and adolescents have not been addressed. Hence, a paucity of studies among children and adolescents in Ethiopia necessitates investigating contextual factors to predict PA and genders disparity. This study, therefore, is aimed at assessing correlates of and gender differences in the level of PA among children and adolescents.

## Methods and Materials

### Study Setting and Sample

This was an observational population-based cross-sectional study conducted in representative samples of school-aged children and adolescents in Addis Ababa between May 2017 and July 2017. The study was conducted in selected sub-cities in Addis Ababa; namely, Bole, Gulele, Kolfe Keranio, Niffasilk Lafto, and Yeka. Hosting 30% of the urban population of Ethiopia, Addis Ababa is considered to be the diplomatic capital of Africa and is one of the fastest growing cities on the continent. All Ethiopian population groups are represented within Addis Ababa due to its position as the capital of the country (23).

The source of data, sample size, and sampling procedure for this study are described and discussed in detail elsewhere (24). Here, we briefly discussed the parameters as follows. The source population was mother-child pairs at the household level living in each sub-city during the study period. The study population was paired sample school-aged children with their mothers present during the data collection period in the selected sub-cities with the inclusion criteria of those children who are living with their mothers, children who are of school age (5–18 years old), mothers who can respond to the interviewer, and school-aged children with their mothers who lived in each of the sub-cities for at least 5 years.

The sample size was calculated using single proportions sample size formula by using Epi Info (Centers for Disease Control and Prevention, Atlanta, GA, USA, 2010) statistical package. The following parameters were used to calculate the sample size: the proportion of children who were overweight in the population (P) is 9.5% (25), 95% CI [the standard normal value at (100%–α) confidence level], d- 3% of Margin of error for sampling and 80% power. This gave a sample size of 367. Then, by including 15% for non-response rate and design effect of 1.5, the total sample size calculated was 634. Multi-stage sampling techniques were carried out to identify the study participants from selected sub-cites. From each sub-city, the proportion to population sampling was applied to obtain the sample size. A simple random sampling method was used to select districts in each sub-city. One child was selected from single-child households, and in some instances, random selection of one child was done when the number of children in the household is greater than one. In this case, a child was selected randomly using the lottery method. In case of the absence of a qualified child in the selected household, the next household was considered.

### Variables and Measurements

The outcome variables used for analysis in this study met the recommended moderate PA (MPA) and moderate-to-vigorous PA (MVPA) in children and adolescents. MPA is described as 60 min or more per day of either moderate or vigorous intensity aerobic PA and includes vigorously intense PA on at least 3 days a week and muscle-strengthening PA (MSPA) as part of 60 min or more of daily PA. Children and adolescents should include muscle-strengthening PA at least 3 days a week (26).

The independent variables in this study were the age group of the children and adolescents, sex of household head, maternal education, maternal occupation, family size, availability of vehicle for family transport, and the type of school where the children attend. Other covariates include the presence of PA sessions and plans and physical education in the school and lifestyle/habit-related factors that are linked with the outcome variable. Sleep duration is categorized as follows: <9, 9–10, and ≥10 h (27), based on the amount of sleep a child gets on a typical school night.

### Data Collection Procedure

Data were collected using a structured questionnaire developed after an in-depth review of literature and adoption of standardized scales (28). The questionnaire, originally prepared in English, was translated into Amharic and retranslated back to English to check and maintain its consistency. Trained data collectors collected the data after it was pre-tested.

A team of interviewers was assigned to each selected sub-city, which consisted of one team supervisor, two female and two male interviewers. Two interviewers were assigned per household, and the supervisors oversaw the coordination aspect of data collection. Data were collected at the household level in the attendance of mothers and children together at their place of residence.

### Data Analysis and Processing

Data were entered using SPSS version 21, and analysis was carried out using STATA 15.0 (Stata Corporation, College Station, TX, USA) and WHO AnthroPlus software v1.02 (WHO, Geneva, Switzerland). Descriptive statistics were used to describe the relationship between meeting the recommended MPA and MVPA with background, household, child, and maternal characteristics of participants. Variables that showed significant association using a liberal *p* < 0.2 in the univariable analysis were included in the multivariable regression. Stepwise multivariable logistic regression models with robust estimation of standard errors were fitted to determine the association. At the final parsimonious model, those variables that retained a *p* < 0.05 were considered to be statistically significant and used to interpret the study findings.

## Results

A total of 632 children and adolescent-parent dyads were included in the study. About 48% were boys and the mean (SD) of the children's age was 12.5 (±2.96) years. Around three-quarters of children and adolescents live in a male-headed household in which the majority of their mothers had formal education, and almost two-thirds were either employed or engaged in a private business. Moreover, around half of the children reside in a household with ≥5 members (Table 1).

**Table 1:** Socio-demographic, economic, and household characteristics of participants by gender in Ethiopia.

**Characteristics/variables**	**Categories**	**Boy *n* (%)**	**Girl *n* (%)**	***P*** **-value**
Age group of the children and adolescent, years	5–9	64 (20.92)	61 (18.71)	0.725
	10–14	152 (49.67)	152 (52.45)	
	15–18	90 (29.41)	94 (28.83)	
Sex of household head	Male	242 (79.08)	242 (73.31)	0.089
	Female	64 (20.92)	87 (26.69)	
Maternal education	No-formal education	119 (38.89)	124 (38.04)	0.826
	Formal education	187 (61.11)	202 (61.96)	
Maternal occupation	Unemployed	103 (33.66)	108 (33.13)	0.658
	Private business	65 (21.24)	61 (18.71)	
	Employed	138 (45.10)	157 (48.16)	
Family size	<5	159 (52.13)	161 (49.39)	0.491
	≥5	146 (47.87)	165 (50.61)	
Presence of vehicle for family transport	Yes	76 (24.84)	83 (25.46)	0.857
	No	230 (75.16)	243 (74.54)	
Type of school where the child attend	Private	163 (53.27)	175 (53.68)	0.917
	Public	143 (46.73)	151 (46.32)	
Childhood overweight and/or obesity	Normal weight	221 (72.22)	229 (70.25)	0.583
	Overweight or obesity	85 (27.78)	97 (29.75)	
Muscle strengthening physical activity (MSPA)	<3 days/week	262 (85.62)	291 (89.54)	0.135
	≥3 days/week	44 (10.46)	34 (10.46)	
Moderate physical activity (MPA)	<3 days/week	254 (83.01)	287 (88.31)	0.057
	≥3 days/week	52 (16.99)	38 (11.69)	
Reported hours of sleep on a typical night	<9 h/day	176 (59.86)	186 (59.81)	0.983
	9–10 h/day	100 (34.01)	107 (34.41)	
	≥10 h/day	18 (6.12)	18 (5.79)	
Reported hours of sleep on a school night	<9 h/day	190 (62.09)	209 (64.31)	0.210
	9–10 h/day	71 (23.20)	83 (25.54)	
	≥10 h/day	45 (14.71)	33 (10.15)	
Received education on how to develop a physical fitness plan	Yes	198 (64.71)	208 (64.00)	0.853
	No	108 (35.29)	117 (36.00)	
Received education about benefits of Physical activity	Yes	220 (71.90)	235 (72.31)	0.908
	No	86 (28.10)	90 (27.69)	
Child participated in physical education in school	Yes	252 (82.35)	263 (80.67)	0.587
	No	54 (17.65)	63 (19.33)	

Although, no disparity was observed with regard to muscle-strengthening activities between boys and girls (10.5% vs. 10.5%, *p* = 0.135), more boys than girls (17.0 and 11.7%) had moderate intensity PA 3 days a week or more (*p* = 0.057). Regarding physical education lessons, more than a quarter and one-third of children were not taught in any of their classes about the benefit of PA and to develop physical fitness plans for themselves, respectively. Further, a significant proportion of the children (39.9% boys and 37.2% girls) was not taught about PA opportunities in their community (Table 1).

The current study found that attending public schools was positively associated with MVPA in girls (adjusted odds ratio [AOR] (95% CI) 3.99 (1.41; 11.3)). Furthermore, an inverse association was found between overweight/obesity and MVPA in the overall children [AOR 95% CI = 0.38 (0.17; 0.87)] and also in sub-samples of girls [AOR (95% CI) 0.14 (0.03; 0.63)]. For MPA, the age of the children, maternal education and occupation, school type, overweight/obesity, and sleep duration on school nights were significant correlates in the studied children.

After adjusting for confounding in the final parsimonious multivariate analysis, late age of children and adolescents, mothers working in a private business, attending public schools, longer sleep duration on school nights, and being taught the benefits of PA were positively associated with meeting MVPA in both sexes combined and in a sub-sample of boys (Table 2). Regarding correlates of MVPA, combined participants in both sexes, adolescents with the age of 15–18 had significantly higher odds of meeting the MVPA than younger children aged 5–9 years [AOR (95% CI) = 9.32 (2.93, 29.6)]. Similarly, children whose mothers had formal education and work in a private business had a significantly higher chance of meeting the MVPA. On the other hand, children who attended public schools also had 3.3 times higher odds of meeting the MVPA, *p* < 0.05. Conversely, overweight/obese children and those who were not taught the benefits of PA had a lesser chance of meeting the MVPA than those who had normal weight and took the lesson, respectively (Table 2).

**Table 2:** Final model of correlates associated with meeting the moderate-to-vigorous physical activity (MVPA) in children and adolescents in Ethiopia.

**Characteristics/variables**	**Categories**	**Both**		**Boys**		**Girls**	
		**Adjusted odd ratio**	***P*** **-value**	**Adjusted odd ratio**	***P*** **-value**	**Adjusted odd ratio**	***P*** **-value**
Age group of the children and adolescent (in years)	5–9	Ref		Ref		Ref	
	10–14	2.78 (0.89; 8.74)	0.079	5.39 (1.01; 28.7)	0.048	1.32 (0.25; 6.85)	0.741
	15–18	9.32 (2.93; 29.6)	<0.0001	28.5 (4.96; 164.2)	<0.0001	3.84 (0.74; 19.9)	0.109
Sex of household head	Male	Ref					
	Female	0.80 (0.40; 1.62)	0.539				
Maternal education	No-formal education	Ref					
	Formal education	2.26 (1.13; 4.48)	0.020				
Maternal occupation	Unemployed	Ref		Ref		Ref	
	Private business	3.65 (1.56; 8.53)	0.003	3.24 (1.10; 9.51)	0.032	2.42 (0.55; 10.6)	0.242
	Employed	1.29 (0.59; 2.83)	0.516	0.83 (0.29; 2.34)	0.719	2.97 (0.86; 10.2)	0.085
Family size	<5	Ref		Ref		Ref	
	≥5	0.55 (0.30;1.02)	0.058	0.45 (0.19; 1.05)	0.065	0.66 (0.25; 1.73)	0.400
Type of school where the child attend	Private	Ref		Ref		Ref	
	Public	3.30 (1.71; 6.35)	<0.0001	2.47 (1.03; 5.92)	0.043	3.99 (1.41; 11.3)	0.009
Childhood overweight and/or obesity	Normal weight	Ref		Ref		Ref	
	Overweight or obesity	0.38 (0.17;0.87)	0.023	0.79 (0.27; 2.29)	0.660	0.14 (0.03; 0.63)	0.011
Reported hours of sleep on a typical night	<9 h/day	Ref		Ref		Ref	
	9–10 h/day	0.44 (0.21;0.92)	0.030	0.71 (0.27; 1.89)	0.495	0.33 (0.10; 1.10)	0.072
	≥10 h/day	0.08 (0.01;0.48)	0.006	0.04 (0.003; 0.49)	0.013	0.11 (0.01; 1.42)	0.091
Reported hours of sleep during school night	<9 h/day	Ref		Ref		Ref	
	9–10 h/day	0.54 (0.24; 1.22)	0.141	0.36 (0.10; 1.26)	0.109	0.81 (0.25; 2.68)	0.736
	≥10 h/day	2.59 (1.08; 6.26)	0.034	2.83 (0.88; 9.09)	0.081	3.12 (0.72; 14.1)	0.127
Received education about benefits of Physical activity	Yes	Ref		Ref		Ref	
	No	0.12 (0.03;0.52)	0.005	0.08 (0.01; 0.47)	0.006	0.35 (0.02; 5.43)	0.453
Child participate in physical education in school	Yes	Ref		Ref		Ref	
	No	0.55 (0.21; 1.45)	0.230	0.43 (0.10; 1.78)	0.243		

Among the sub-sample of boys, adolescents aged 10–14 and 15–18 years had significantly higher odds of meeting the MVPA than the younger ones (5–9 years). Parallel to the results of both sexes combined, those whose mothers work in a private business and who attended public schools have higher odds of meeting the MVPA. Besides, those who were sleeping ≥10 h on a typical night and who were not taught the benefits of PA were less likely to meet the MVPA compared to their counterparts (Table 2). On the other hand, among the sub-sample of girl's children, only those attending public schools and overweight/obese children were more likely to meet the MVPA than private school and normal weight children. Age, maternal occupation, and being taught the benefits of PA did not significantly predict MVPA unlike the results in boys (Table 2). Regarding MPA; age, maternal education and occupation, school type, overweight/obesity, and sleep duration on school nights were significant correlates in the studied participants. Age, school type attended, and overweight/obesity were also significant correlates of MPA in boys. Furthermore, family size and school type attended were the significant correlates of MPA in girls. Children who were living in families ≥5 members were less likely to meet the MPA than those in families <5 members (Table 3).

**Table 3:** Final model of correlates associated with meeting the moderate physical activity (MPA) in children and adolescents in Ethiopia.

**Characteristics/variables**	**Categories**	**Both**		**Boys**		**Girls**	
		**Adjusted odd ratio**	***P*** **-value**	**Adjusted odd ratio**	***P*** **-value**	**Adjusted odd ratio**	***P*** **-value**
Age group of the children and adolescent (in years)	5–9	Ref		Ref		Ref	
	10–14	2.91 (1.05; 8.04)	0.039	5.35 (1.09; 26.3)	0.039	1.74 (0.42; 7.29)	0.447
	15–18	5.99 (2.12; 16.9)	0.001	14.0 (2.82; 69.5)	0.001	2.47 (0.56; 10.8)	0.231
Sex of household head	Male						
	Female						
Maternal education	No-formal education	Ref				Ref	
	Formal education	1.96 (1.06; 3.64)	0.033			2.20 (0.73; 6.60)	0.159
Maternal occupation	Unemployed	Ref		Ref		Ref	
	Private business	2.51 (1.11; 5.69)	0.027	2.52 (0.91; 6.95)	0.075	2.28 (0.46; 11.4)	0.316
	Employed	1.72 (0.84; 3.50)	0.134	1.30 (0.53; 3.22)	0.569	3.25 (0.91; 11.7)	0.070
Family size	<5	Ref				Ref	
	≥5	0.60 (0.35; 1.06)	0.077			0.28 (0.10; 0.75)	0.011
Presence of vehicle for family transport	Yes	Ref				Ref	
	No	1.22 (0.60; 2.51)	0.580			1.15 (0.35; 3.76)	0.814
Type of school where the child attend	Private	Ref		Ref		Ref	
	Public	3.76 (1.95; 7.25)	<0.0001	3.73 (1.69; 8.22)	0.001	3.85 (1.33; 11.1)	0.013
Childhood overweight and/or obesity	Normal weight	Ref		Ref		Ref	
	Overweight or obesity	0.33 (0.16; 0.70)	0.004	0.28 (0.09; 0.83)	0.021	0.35 (0.12; 1.04)	0.059
Reported hours of sleep on a typical night	<9 h/day	Ref		Ref		Ref	
	9–10 h/day	0.42 (0.21;0.84)	0.014	0.52 (0.21; 1.29)	0.160	0.45 (0.16; 1.26)	0.129
	≥10 h/day	0.30 (0.08; 1.16)	0.082	0.49 (0.08; 3.00)	0.444	0.10 (0.01; 1.24)	0.073
Reported hours of sleep during school night	<9 h/day	Ref		Ref		Ref	
	9–10 h/day	0.54 (0.25; 1.15)	0.111	0.54 (0.19; 1.54)	0.249	0.47 (0.15; 1.48)	0.197
	≥10 h/day	2.35 (1.02; 5.41)	0.045	2.53 (0.81; 7.89)	0.109	2.24 (0.57; 8.87)	0.249
Received education on how to develop a physical fitness plan	Yes	Ref		Ref		Ref	
	No	1.67 (0.48; 5.73)	0.417	1.03 (0.20; 5.24)	0.969	4.15 (0.38; 45.4)	0.244
Received education on benefits of physical activity	Yes	Ref		Ref		Ref	
	No	0.43 (0.14; 1.35)	0.149	0.34 (0.08; 1.47)	0.149	1.45 (0.13; 15.8)	0.761
Child participates in physical education in school	Yes	Ref		Ref		Ref	
	No	0.47 (0.19; 1.14)	0.095	0.39 (0.10; 1.50)	0.169	0.72 (0.21; 2.55)	0.616

## Discussion

Our study revealed that most children did not meet the daily recommended PA. In agreement with our finding, a pooled analysis conducted in 146 low-income countries found that among children aged 11–17 years insufficient PA was observed in about 85% of the participants (1). Similarly, another study in Ethiopia showed a comparable proportion; only 17.2% children were physically active (29). Our study strongly supports that boys were more active than boys in which there was a 5.3% difference in the proportion of MPA ≥ 3 days a week. This finding seemed to be consistent with most of the studies reviewed (1, 13, 29–35). Though the criteria of classifying as active/in-active differed across studies; Regina et al. and Gerson et al. reported 7.1 up to 38.8 percentage point increment in boys MVPA than girls (1, 30). Likewise, 20.6% of more boys also participated in PA ≥5 days/week in a study in the US (31). Further, another study in Brazilian children and a review paper using cluster analysis also showed that boys spend significantly higher time in PA than girls (13, 32). As reported in the review paper mentioned before (13), girls tended to engage more in homework and/or socializing through the phone which could be one reason and they were also more likely to report the feeling that they did not enjoy PA (33). Other studies also indicated that the lowest prevalence in female insufficient activity can be potentially explained by societal factors, such as girls being required to support activity and domestic chores around the home in south Asia countries (1), which is a similar feature with this study. The constant finding that boys engage more in both moderate and vigorous PA underlines the need for PA intervention programs to target girls of all ages.

In contrary to previous studies, in both sexes combined, and sub-sample of boys alone, we observed that older children (15–18) significantly engage in MVPA and MPA than younger ones (5–9 years). A review by Rebecca revealed that older children tend to be in clusters defined by low levels of PA. It was gender specific in two of the studies reviewed that older boys in one study and older girls in the other were observed with low levels of PA (13). Other studies in Nigeria and Ethiopia also evidenced MPA, MVPA, and total PA were lower in older adolescents and PA level decreased as age increased among girls in a study in Senegal (29, 34, 36). The disagreement could be attributed to the fact that most of the studies included older children (9–21, 14–19, 12–18, and 13–16 years, respectively) and one is conducted among girls only in whom no association was found between girls' age and PA in our study. Though it is conducted among young children, Dias et al. reported that older ones spent more time in MVPA than younger children similar to the current study (37). However, the association between age and PA may not be conclusive.

The present study also found that children whose mothers worked in a private business had higher odds of meeting both the MVPA and MPA compared to children with unemployed mothers. In line with our finding, Ferrari et al. found that children whose mothers worked full-time had more MVPA than those whose mothers worked part-time in Brazil (30). Another study also mentioned that screen time of children increased when mothers were unemployed (20) but there was no association with PA (20, 38). Though it is a distal indicator, practical family support, such as providing the necessary sports facilities and transportation to get to a place where children can do PA, was associated with a higher level of PA (38) in which mothers working in a private business can do better than unemployed ones. However, a better understanding is needed on how a mother's employment affects PA.

The present study also found that children attending public schools were more likely to meet both MVPA and MPA than children in private schools. This finding is supported by other studies in India, Brazil, and Addis Ababa, which reported higher PA level among government school children (39–41). No association was also reported by Ferrari et al. (30). The results in the current study could be explained as, in Ethiopia, children in private schools tend to be from families of higher socio-economic status and spend their time in more sedentary activities, such as longer screen time and a better access to transport facilities. In the other way, public school children, who mostly are from lower socio-economic status families, also tend to involve in household chores that increase their level of PA than children in private schools (42).

In this study, in both sexes combined, overweight/obesity was inversely associated with both MVPA and MPA. Further MVPA is associated with girls and MPA with boys. Though it is not possible to convey causality because of the cross-sectional nature of the current and most reviewed studies, comparably, anthropometric variables (waist circumference, BMI, and body fat percent) have negative association with MVPA and MPA in the studied participants (30, 32, 43). Similarly, MPA and VPA were also negatively associated with body composition variables (BMI and body fat percent) in boys and girls, respectively (32). Studies that did not consider the intensity of PA also found similar results. A review with cluster analysis study mentioned that low PA characterized clusters showed a positive association with overweight and another study reported those in low PA cluster had the highest odds of being overweight (13). Moreover, substantial associations between overweight/obesity and low levels of PA also showed among studies in Africa and Ethiopia as PA burns off body fat and hence prevents obesity (44–47).

Moderate-to-vigorous physical activity and MPA were associated with sleep duration on typical and school nights negatively and positively in both sexes combined, respectively. A similar association was also found for MVPA in boys. Consistent findings are available in other studies as longer sleep contributes to an increase in PA level in both sexes combined (48). Lindsay et al. also found early onset and longer sleep duration were associated with increased MVPA (49). This could also explain the negative association on typical nights that may be attributed to late onset. Insufficient sleep duration has been shown to be associated with excessive TV watching, morning tiredness, and reduced odds of participating in PA in both boys and girls (50). Conversely, Sleep quantity was found not to be associated with PA (30). Studies reviewed considered the sleep duration in any nights, however, most of the nights of children are supposed to be school nights.

Physical education has become convenient and inexpensive way of fostering PA and fitness, which can reach large numbers of children across various demographic groups. In this study, being taught the benefits of PA was associated with higher odds of meeting the MVPA in both sexes combined and boys only. An intervention study showed that the mean PA score of the students significantly increased after a 1-month of educational intervention in terms of knowledge (51). Another review in secondary school physical education classes also reported that adolescents were least active when physical education lesson context was knowledge which may result in the small amount of MVPA time (21). The differences demonstrated that teachers, parents, and coaches need to consider gender differences in mixed physical education and sports settings because activities that focus on physical performance are likely to favor boys and teachers, in particular, need to know how to conduct PE and sports that provides boys and girls with equal opportunities for sustained engagement, development of competency and enjoyment of PA (6). However, it included secondary school students in which behavioral change may be less likely than younger children and adolescents. Moreover, in the current study, being taught the benefits of PA did not necessarily mean children spend PE classes with knowledge context only.

The strengths of this study include the inclusion of a wide range of the children age group and a large number of analyzed covariates, considering gender differences for MVPA and MPA levels. In addition, the study may be one of the few similar studies conducted in Ethiopia, which can be considered as added strength. The results of this study may have been influenced by recall bias since the results are based on self-reported PA level. Using reported PA levels, rather than objective measurement, may have limited validity in measuring PA to some extent. Furthermore, there may be information bias in the reporting of PA and sleep duration by the parent for the children. The cross-sectional design of the study precludes us from conclusively making causal inferences and not covering a wide geographic area is also worth mentioning.

## Conclusion

The present study revealed that older age of children and adolescents, mothers working in a private business, attending public schools, longer sleep duration on school nights, and being taught the benefits of PA were positively associated with meeting MVPA in both sexes combined. Furthermore, the age of the children, maternal education and occupation, school type, overweight/obesity, and sleep duration on school nights were significant correlates in both sexes combined for MPA. Our study found that girls are less likely than boys to engage in PA with more than 5 percentage point difference Comprehensive multifaceted health behavior modification and interventions, such as facilities for PA through the provision of conducive school environment that includes sport and recreational services would encourage children to engage in PA more, and hence reduce the risks at later ages associated with overweight and obesity. Regular physical education on the benefits of PA in school needs to be strengthened as well. Future studies should focus on objective PA measurement in a larger and more diverse representative samples. The cross-sectional nature of this study precludes our ability to conclusively decide on correlates of PA. As a result, longitudinal studies with a long follow-up period would also provide more evidence on correlates of PA.

## Data Availability Statement

The original contributions presented in the study are included in the article/supplementary material, further inquiries can be directed to the corresponding author/s.

## Ethics Statement

Ethical approval was obtained from the Department of Health Studies, University of South Africa Ethical Clearance Committee for Research on Human Subjects (HSHDC/ 575/2016) and Addis Ababa City Administration Health Bureau (A/A/H/B/3542/227). Information about the study was given to the participants including purposes as well as potential risks and benefits rendered. Official letters of co-operation from the above organizations were given to respective sub-cities and District administrators. Informed consent was obtained from the participants' parents. For those children under the age of 18 years, a letter of assent form was developed for participation and obtained from their parents. Written informed consent to participate in this study was provided by the participants' legal guardian/next of kin.

## Author Contributions

SB conceived the idea, wrote and designed the protocol, data management and analysis, drafted the manuscript, and participated in the critical revision of the manuscript. BG, TM, and AA analysis and interpretation of data, drafted the manuscript, and participated in the critical revision of the manuscript. All authors read and approved the final manuscript.

## Conflict of Interest

The authors declare that the research was conducted in the absence of any commercial or financial relationships that could be construed as a potential conflict of interest.

## Publisher's Note

All claims expressed in this article are solely those of the authors and do not necessarily represent those of their affiliated organizations, or those of the publisher, the editors and the reviewers. Any product that may be evaluated in this article, or claim that may be made by its manufacturer, is not guaranteed or endorsed by the publisher.
